# A Phase II Study of ERK Inhibition by Ulixertinib (BVD-523) in Metastatic Uveal Melanoma

**DOI:** 10.1158/2767-9764.CRC-24-0036

**Published:** 2024-05-21

**Authors:** Elizabeth I. Buchbinder, Justine V. Cohen, Giuseppe Tarantino, Christine G. Lian, David Liu, Rizwan Haq, F. Stephen Hodi, Donald P. Lawrence, Anita Giobbie-Hurder, Deborah Knoerzer, Ryan J. Sullivan

**Affiliations:** 1 Department of Medical Oncology, Dana-Farber Cancer Institute, Boston, Massachusetts.; 2 Department of Medicine, Brigham and Women's Hospital, Boston, Massachusetts.; 3 Harvard Medical School, Boston, Massachusetts.; 4 Department of Pathology, Brigham and Women's Hospital, Boston, Massachusetts.; 5 Department of Medical Oncology, Massachusetts General Hospital, Boston, Massachusetts.; 6 Division of Biostatistics, Department of Data Science, Dana-Farber Cancer Institute, Boston, Massachusetts.; 7 BioMed Valley Discoveries, Kansas City, Missouri.

## Abstract

**Purpose:**

Uveal melanoma is a rare and aggressive subset of melanoma that is minimally responsive to traditional therapies. Greater than 80% of uveal melanomas have a mutation in GNAQ or GNA11 which lead to downstream signaling through the MAPK pathway. Ulixertinib (BVD-523) is a potent and reversible small-molecule ATP-competitive inhibitor of both ERK1 and ERK2 protein kinases.

**Materials and Methods:**

We performed a phase II study to determine the efficacy and safety of BVD-523 in patients with metastatic uveal melanoma. This was conducted as a Simon two-stage design with a sample size of 25 patients and an initial evaluation of efficacy after 13 patients.

**Results:**

From April 2018 to April 2019, 13 patients were enrolled. Patients were predominantly female (69%) with a median age of 64 years (34–76). Sites of metastases included liver (84.6%) and lung (30.8%). Grade 3 and 4 toxicities associated with therapy were consistent with ERK inhibitors and included liver function test (LFT) elevation, hyponatremia, pruritis, amylase elevation, anemia, and rash. The best response, per RECIST 1.1, was stable disease in 4 patients, and disease progression in 7 patients. Two patients were unevaluable for response due to withdrawal from study. Median time to progression was 2.0 months. There were eight deaths due to disease progression with a median overall survival of 6.9 months.

**Conclusions:**

ERK inhibition with ulixertinib (BVD-523) did not demonstrate activity in patients with metastatic uveal melanoma. The toxicities observed were consistent with what would be expected with MAPK pathway inhibition.

**Significance:**

Uveal melanoma is a difficult to treat disease with minimal therapy options. The majority of uveal melanomas have mutations in GNAQ or GNA11 leading to activation of the MAPK pathway. Efforts to target MEK in uveal melanoma has had mixed results. This phase II trial of ERK inhibition with BVD-523 examined the potential role of this agent in uveal melanoma therapy.

## Introduction

In 2023 it is estimated that approximately 97,610 new cases of malignant melanoma will be diagnosed in the United States, 5% of these, approximately 2,000 cases will be uveal melanoma (1, 2). Uveal melanoma is a rare malignancy that arises from the iris, ciliary body, or choroid of the eye. Despite the ability to achieve local control in the majority of patients, a quarter of patients will die of metastatic disease (3). The most common sight of initial metastasis is the liver due to hematogenous spread with an average survival of approximately 1 year after metastatic disease is diagnosed (4). Management of uveal melanoma differs from that of cutaneous melanoma, as inconsistent responses are seen to the therapies used most commonly for cutaneous melanoma, including ipilimumab, the anti-PD1 antibodies nivolumab and pembrolizumab, and chemotherapy. Combination immunotherapy with ipilimumab and nivolumab is often used with a response rate of only around 12% (5). Recently, there was an advance in the treatment of uveal melanoma with tebentafusp, a bispecific molecule targeting gp100 demonstrating prolonged survival (6, 7). The survival benefit observed with the use of tebentafusp in patients with uveal melanoma who were found to be HLA-A*0201 positive occurred without robust imaging responses; however, the 3-year update shows that only a minority of patients survive 3 years (2023 NEJM). Despite this new therapy, there is still a large need for additional treatments for patients with uveal melanoma.

Unlike cutaneous melanoma, BRAF mutations are rarely (if ever) observed in uveal melanoma. However, greater than 80% of uveal melanomas have activating mutations in *GNAQ* or *GNA11*, genes that encode for G protein alpha subunits (8–10). *GNAQ* and *GNA11* mutations lead to downstream signaling through the MAPK pathway. This has led to attempts at therapeutic targeting of the MAPK pathway with MEK inhibition in patients with uveal melanoma. In a randomized phase II trial, 99 patients were randomized to receive the MEK inhibitor selumetinib or chemotherapy (investigator's choice of dacarbazine or temozolomide). Treatment with selumetinib was associated with a progression-free survival (PFS) of 15.9 versus 7 weeks with chemotherapy, and objective response rates of 14% and 0%, respectively (11). Analysis of patients treated on this study supported a MAPK dependence which was reflected in MEK-dependent gene expression changes in response to therapy (12). Based upon this a phase III, double blind study comparing selumetinib plus dacarbazine versus dacarbazine alone was performed. Unfortunately, in this study there was no improvement of PFS observed with the addition of selumetinib (13).

The ERK family kinases are MAPK signaling components downstream of MEK1/2, and ERK activation in uveal melanoma has been observed in relation to GNAQ and GNA11 mutations (8, 9). In addition, ERK inhibition has been seen to overcome acquired resistance to MEK inhibitors in preclinical studies (14). BVD-523 is a small-molecule inhibitor of ERK kinase that has completed phase I testing and has demonstrated activity in patients with BRAF/MEK-inhibitor refractory, BRAF-mutant melanoma as well as in a patient with NRAS-mutant melanoma with a reasonable safety profile (15). Given the role of the MAPK pathway on development and growth of uveal melanoma and the initial activity in other MAPK-dependent melanoma settings, we performed a phase II clinical trial examining BVD-523 in patients with metastatic uveal melanoma.

## Materials and Methods

### Study Design

This phase II study planned to enroll up to 25 patients with metastatic uveal melanoma to evaluate the efficacy of BVD-523. In the first stage, 13 patients were enrolled at which time an initial evaluation for efficacy was performed. The protocol was approved by the Dana-Farber/Harvard Cancer Center (DF/HCC) Institutional Review Board. The study investigators obtained written informed consent from the patients prior to performing any study-specific procedures. The study was conducted in accordance with the Declaration of Helsinki, CIOMS, Bellmont Report and U.S. Common Rule. This trial was listed on clinicaltrials.gov, NCT03417739.

### Patient Selection

Patients were eligible if they had histologically or cytologically confirmed stage IV uveal melanoma, measurable disease using RECIST 1.1 and were Eastern Cooperative Oncology Group (ECOG) performance status (PS) 0 to 2. Patients could have received any number of prior therapies excluding prior ERK inhibition. Patients were excluded if they had active brain metastasis or history of current evidence/risk of retinal vein occlusion or central serous retinopathy. LFTs were required to be ≤2.5 × institutional upper limit of normal, unless there was known liver involvement in which case ≤5.0 × institutional upper limit of normal.

### Procedures

Patients received BVD-523 (600 mg) twice daily orally for 28 consecutive days at 12 ± 2 hour intervals. The treatment cycle was defined as 28 days. Treatment was continued until objective disease progression, intolerable toxicity, or other discontinuation criteria were met. Patients were assessed by CT scan or MRI every 8 weeks for tumor assessment and response was graded by RECIST. Toxicity and adverse events (AE) were recorded from the time of informed consent and graded by CTCAE 4.03.

### Statistical Analysis

The primary endpoint of the study was the overall response rate. ERK inhibition therapy would have been considered effective in the treatment of uveal melanoma if a 25% response rate was observed. A Simon two-stage design was performed comparing a null rate of 5% against an alternative rate of 25%. Thirteen patients were enrolled into the first stage of the trial and if one or fewer responses were observed the trial would be terminated for lack of efficacy. If two or more responses were seen, an additional 12 patients would have been enrolled into the second stage of the trial. If a total of 3 or more of the 25 total patients had a response the treatment would be considered successful. The trial was designed with a one-sided type 1 error of 8% (target 10%) and 87% power (target 85%). If the BVD-523 was not superior to selumetinib (and the true response rate is 5%) then the probability was 0.86 of stopping at the end of the first stage.

### NanoString Analysis

Tumor biopsies were obtained prior to the first treatment and 12–16 days following the initial treatment. Biopsies were paraffin embedded and stored for subsequent testing. NanoString testing was performed by standard procedures with the Tumor Signaling 360 panel. Quality control (QC) analysis was performed evaluating the NanoString nCounter quality control metrics. We assessed the overall performance of the nCounter assay by evaluating the imaging and binding density QC metrics, and by assessing the performance of the positive controls using the positive control linearity and limit of detection parameters. In addition, we performed an overall visual inspection of the data and assessed the severity of any QC flags. No flags were identified in the samples analyzed. The standard normalization steps recommended by NanoString were performed. Specifically, we adopted the following approaches:

- Positive Control Normalization: Where the geometric mean of positive control probes was used to calculate a scaling factor for each sample.
- Background Assessment: Negative control probes were utilized to assess whether endogenous genes are expressed above the level of background.
- Housekeeping Normalization: The geometric mean of housekeeping gene expression was used to calculate a scaling factor for each sample.

To assess the performance of the normalization procedure, we evaluated the relative log expression. Comparison between pretreatment and on-treatment was performed using R, with the packages *limma* and *NanoStringDiff*. Subsequently, gene set enrichment analysis (GSEA) was performed with the R library *genekitr*. Finally, the individual normalized expression of DUSP6 and the signature score of the MAPK genes were compared between pretreatment and on-treatment timepoints using a Wilcoxon paired test.

### Data Availability

The data generated in this study are not publicly available due to patient confidentiality but are available upon request from the corresponding author at elizabeth_buchbinder@dfci.harvard.edu

## Results

### Patients

The first patient was enrolled on April 23, 2018 and the last patient on April 12, 2019. Six were enrolled from Dana-Farber Cancer Institute (46%) and 7 from Massachusetts General Hospital (54%). Patients were predominantly female (69%) and White (92%); the median age at enrollment was 64 years (range: 34–76 years). Median patient body mass index was 24.4 (range: 19–35); 12 patients (92%) had ECOG PS of 0 or 1. Eleven of the patients had radiation therapy (XRT) to treat their primary uveal melanoma and 10 had surgery. Eight patients received systemic therapy in the metastatic setting (Table 1; Supplementary Table S1).

**TABLE 1:** Baseline characteristics

Baseline characteristic	All patients (*n* = 13)
Sex	
Female	69% (9)
Male	31% (4)
Age (median, range)	64 (34–76) y
ECOG	
0	69% (9)
1	23% (3)
2	8% (1)
LDH	
≤ULN	61.5% (8)
>ULN	38.5% (5)
Prior therapy	
Chemo/IO	62% (8)
Sites of metastatic disease	
Liver	85% (11)
Lung	31% (4)
Peritoneum	15% (2)
Kidney	8% (1)
Spine	8% (1)

Abbreviations: IO, immunotherapy; LDH, lactate dehydrogenase; ULN, upper limit of normal; y, years.

At the time of study enrollment, patients were a median of 7.3 months (range: 0.2–135.8 months) after diagnosis of metastatic disease. Ten patients (77%) had a pretreatment biopsy (liver: 9; chest wall: 1). One patient was tested for genetic mutation and was found to have a *GNA11* mutation.

### Efficacy

**FIGURE 1:**
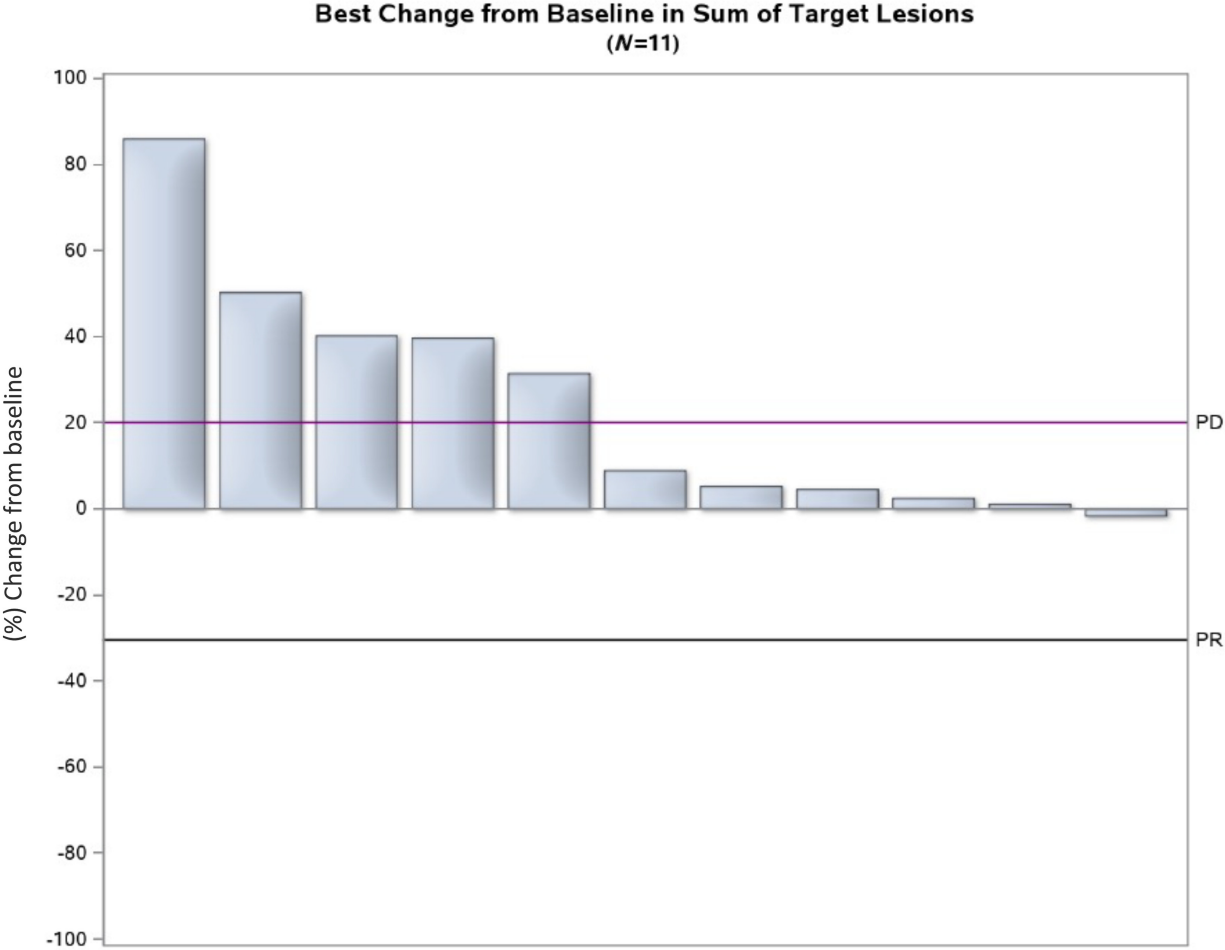
Waterfall plot: depiction of percent tumor change among evaluable patients.

The primary endpoint of overall response was not met. No patients had partial or complete responses as best response to therapy, resulting in a response rate of 0% [90% exact confidence interval (CI): 0–20.5]. Four patients (31%) had best response of stable disease, 7 (54%) had best response of disease progression (PD), and 2 were unevaluable for response (15%; Fig. 1). Thus, according to the Simon two-stage design, the trial stopped recruitment at this point due to lack of responders in the first stage. Ten patients ultimately had PD, with a median time to progression (TTP) of 2.0 months (90% CI: 1.8–3.6 months).

**FIGURE 2:**
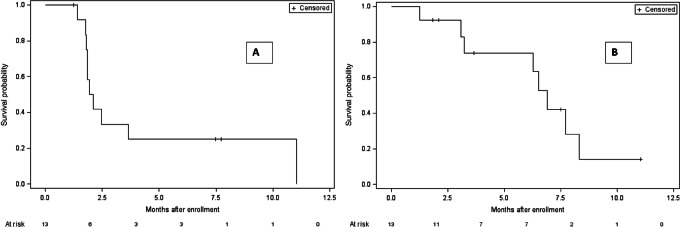
TTP and OS. **A,** Kaplan–Meier estimates of TTP. **B,** Kaplan-Meier estimates of OS.

As of the data retrieval data, all patients were off therapy. Ten patients stopped therapy due to disease progression, 2 for unacceptable toxicity, and 1 due to patient decision. Patients completed a total of 34 treatment cycles (median: 2, range: 1–12). There were eight deaths with a median overall survival (OS) of 6.9 months (90% CI: 3.2–8.3 months; Fig. 2).

### Toxicity

Patients attempted a total of 37 cycles of BVD-523 (median: 2; range: 1–12). There were 15 occurrences of a dose being held during a cycle and four occurrences of dose reduction. All dose modifications were due to toxicity.

The majority of grade 1 and 2 AEs were gastrointestinal disorders with 10 reports of diarrhea, seven reports of nausea, and five reports of abdominal pain (Supplementary Tables S2 and S3). There were also five reports of grade 1 or 2 fatigue and seven reports of loss of appetite. There were also common reports of rash with 11 of the 13 patients experiencing rash, four grade 1 and 2 reports of pruritis, seven of acneiform rash, and three of maculopapular rash. A summary of grade 3 and 4 AEs is provided in Table 2.

**TABLE 2:** Grade 3 and 4 toxicities and AEs, all attributions. Patients may have experienced more than one toxicity

*Toxicity Category CTCAE v4.0*	*Toxicity Description CTCAE v4.0*	Grade 3	Grade 4
All categories	Total	17	3
*Blood and lymphatic system disorders*	*Anemia*	1	—
*Cardiac disorders*	*Supraventricular tachycardia*	1	—
General disorders and admin site conditions	*Fatigue*	1	—
	*Fever*	1	—
*Infections and infestations*	*Urinary tract infection*	1	—
*Injury, poisoning and procedural complications*	*Fall*	1	—
	*Fracture*	1	—
*Investigations*	*Alanine aminotransferase increased*	1	1
	*Aspartate aminotransferase increased*	2	1
	*Serum amylase increased*	1	—
*Metabolism and nutrition disorders*	*Hypoalbuminemia*	1	—
	*Hyponatremia*	—	1
*Respiratory, thoracic and mediastinal disorders*	*Dyspnea*	1	—
	*Hypoxia*	1	—
*Skin and subcutaneous tissue disorders*	*Pruritis*	1	—
	*Rash maculopopular*	1	—
*Vascular disorders*	*Thromboembolic event*	1	—

### Molecular Analysis

**FIGURE 3:**
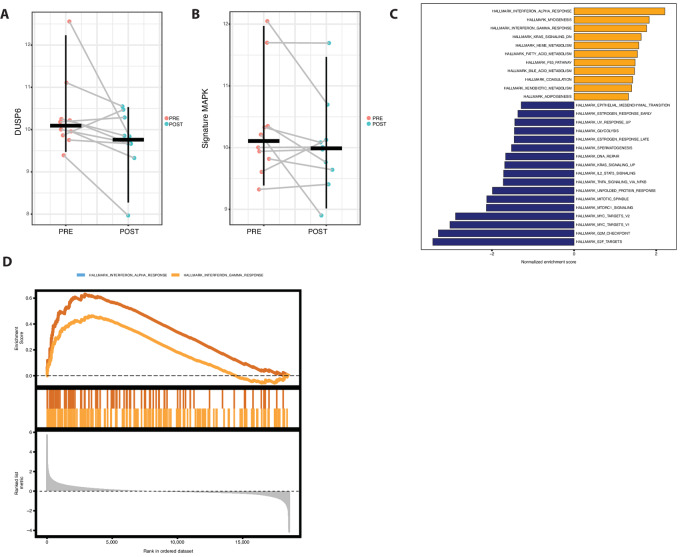
(**A**) NanoString data nSolver normalized log_2_ counts of DUSP6 pretreatment and on-treatment. **B,** MAPK signature evaluated at pretreatment and on-treatment using four genes included in the NanoString panel, specifically DUSP6, MYC, TNFAIP3, and PMAIP1. **C,** GSEA against the Hallmark database; enriched signatures on treatment are depicted in orange, while enriched signatures pretreatment are in blue. **D,** Enrichment scores of two signatures related to IFN gamma and alpha responses, which are enriched in the on-treatment samples.

Pretreatment and on-treatment biopsies were collected from patients who underwent therapy on trial. Although biopsies were planned for all patients sufficient paired biopsies were available on 10 patients. IHC staining for pERK worked well which was not significantly different between pretreatment and on-treatment samples which is not unexpected because ulixertinib raises levels of pERK NanoString analysis of samples pretreatment and on-treatment with ERK inhibition using the Mann–Whitney paired test indicated a nonsignificant reduction of DUSP6 (log_2_FC = 0.59, *P* = 0.08). By applying preranked GSEA to the data from the pretreatment versus on-treatment comparison, we identified an enrichment of KRAS signaling in the pretreatment samples. Conversely, there was a depletion of this signaling in the samples taken during treatment, indicating a potential inhibition of the MAPK pathway (Fig. 3). However, reduction in expression of a signature composed of MAPK genes (MYC, DUSP6, TNFAIP3, PMAIP3, log_2_FC = 0.3, *P* = 0.23), within this limited size cohort, was not seen (Fig. 3A and B). A limited GSEA using cancer hallmark gene sets showed enrichment of IFN gamma and alpha response signatures in on treatment tumors, suggesting an immune response to therapy despite the lack of overall effect (Fig 3C and D). A predose sample taken at steady state (cycle 1 day 15) was drawn for pharmacokinetics on this study and the mean concentration for the steady-state samples was 1,039 ng/mL which is an expected trough level from prior studies. This level also correlated with near complete inhibition of pRSK/total RSK in the blood in prior studies (15).

## Discussion

This study expands upon previous studies examining drugs targeting the MAPK pathway in patients with metastatic uveal melanoma. Prior studies of MEK inhibition with selumetinib single agent were initially promising but subsequently did not demonstrate significant activity against this highly resistant disease in randomized trial; and the response rate of selumetinib with dacarbazine was a disappointing 3% (13).

The toxicities observed on study were consistent with what would be expected with MAPK pathway inhibition including rash, fatigue, nausea, diarrhea, and LFT elevation, suggesting the pathway was targeted appropriately by ulixertinib therapy. NanoString analysis suggested that ERK inhibition was achieved through the downregulation of KRAS and MYC signaling pathways. It also showed a marginal reduction in DUSP6 expression, though not statistically significant (*P* = 0.08). However, one limitation to note is that these findings are derived from bulk sample analysis rather than single-cell resolution, which may obscure specific cellular responses. The NanoString data did demonstrate enrichment of IFN gamma and alpha response signatures in on treatment tumors. These findings suggest that despite a lack of overall antitumor benefit, there may have been an immune response to therapy and therefore, perhaps there may be promise in combining ulixertinib with immunotherapy.

Future studies in uveal melanoma will need to target novel pathways in the disease or consist of combinations which overcome the resistance seen in this disease. There has been some activity observed with immunotherapy in this disease and frontline therapy at this time consists of either tebentafusp in HLA A*201 positive patients or combination immune checkpoint inhibition. A novel therapy combining darovasertib (protein kinase C small-molecule inhibitor) with crizotinib (c-MET inhibitor) has had some promising clinical activity and trials with this combination are ongoing (16). However, uveal melanoma remains a challenging malignancy to treat and many patients will likely need further therapy for their disease.

In this study, ERK inhibition with ulixertinib (BVD-523) similarly did not demonstrate activity in patients with metastatic uveal melanoma. These studies suggest that targeting the MAPK pathway on its own may not be the optimal approach in uveal melanoma despite the high prevalence of GNAQ and GNA11 mutations and resultant MAPK activation observed. Future studies in uveal melanoma will need to target novel pathways and immune mechanisms.

## Supplementary Material

Supplementary table 1 Supplementary table 1: Representativeness of study participants

Supplementary table 2 Supplementary table 2: All adverse events/toxicities regardless of attribution

Supplementary table 3 Supplementary table 3: Adverse Events/toxicities felt to be at least possibly related to drug.
