# Supplementary material for: A Phase II Study of ERK Inhibition by Ulixertinib (BVD-523) in Metastatic Uveal Melanoma
Source: Cancer Res Commun. 2024 May 21;4(5):1321–7. doi: 10.1158/2767-9764.CRC-24-0036 (PMC11107576; doi:10.1158/2767-9764.CRC-24-0036)
Supplement: Supplementary table 1 — Representativeness of study participants [file crc-24-0036-s01.docx]

**Supplemental Data**

**Supplementary Table 1 Representativeness of Study participants**

| **Cancer type: Uveal melanoma** | |
| --- | --- |
| **Considerations related to:** | |
| **Sex** | **Uveal melanoma is an increadibly rare tumor. It has a higher incidence in men than women.** |
| **Age** | **Uveal melanoma is most commonly diagnosed in the sixth decade of life** |
| **Race/ethnicity** | **Uveal melanoma is predominantly found in Caucasians (95% of cases in Caucasians)** |
| **Geography** | **There is no available data on incidence of uveal melanoma outside of North America and Europe** |
| **Overall represantiveness of this study** | **Uveal melanoma is a rare tumor found predominantly in Caucasian populations. Our study enrolled more women than men despite there being a generally higher incidence in men than women. Otherwise the characteristics match the demographics of the disease.** |
