# Supplementary material for: A Phase II Study of ERK Inhibition by Ulixertinib (BVD-523) in Metastatic Uveal Melanoma
Source: Cancer Res Commun. 2024 May 21;4(5):1321–7. doi: 10.1158/2767-9764.CRC-24-0036 (PMC11107576; doi:10.1158/2767-9764.CRC-24-0036)
Supplement: Supplementary table 2 — All adverse events/toxicities regardless of attribution [file crc-24-0036-s02.docx]

**Supplementary Table 2 – Adverse Events/Toxicities (all attributions)**

|  | | *Toxicity Grade CTCAE v4.0* | | | |
| --- | --- | --- | --- | --- | --- |
|  |  | *1* | *2* | *3* | *4* |
|  |  | *N* | *N* | *N* | *N* |
| *Toxicity Category CTCAE v4.0* | *Toxicity Description CTCAE v4.0* | 1 | - | 1 | - |
| *Blood and lymphatic system disorders* | *Anemia* |  |  |  |  |
|  | *Eosinophilia* | 1 | - | - | - |
|  | *Lymph Node Pain* | 1 | - | - | - |
| *Cardiac disorders* | *Palpitations* | 1 | - | - | - |
|  | *Supraventricular Tachycardia* | - | - | 1 | - |
| *Eye disorders* | *Blurred Vision* | 1 | - | - | - |
|  | *Floaters* | 1 | - | - | - |
|  | *Halos And Glowing Around Lights* | 1 | - | - | - |
|  | *Scotoma* | 1 | - | - | - |
| *Gastrointestinal disorders* | *Abdominal Distension* | - | 1 | - | - |
|  | *Abdominal Pain* | 6 | - | - | - |
|  | *Anal Ulcer* | - | 1 | - | - |
|  | *Ascites* | - | 1 | - | - |
|  | *Constipation* | 3 | - | - | - |
|  | *Diarrhea* | 9 | 1 | - | - |
|  | *Epigastric Pain* | 2 | - | - | - |
|  | *Esophageal Stenosis* | 1 | - | - | - |
|  | *Gastroesophageal Reflux Disease* | - | 1 | - | - |
|  | *Gingival Pain* | 1 | - | - | - |
|  | *Hemorrhoids* | - | 1 | - | - |
|  | *Mouth Sores* | 1 | - | - | - |
|  | *Mucositis Oral* | 1 | - | - | - |
|  | *Nausea* | 5 | 3 | - | - |
|  | *Rectal Ulcer* | 1 | - | - | - |
|  | *Stomach Pain* | 1 | - | - | - |
|  | *Vomiting* | 1 | 1 | - | - |
| *General disorders and admin site conditions* | *Chills* | 1 | - | - | - |
|  | *Dry Heaves* | - | 1 | - | - |
|  | *Edema Limbs* | 4 | - | - | - |
|  | *Fatigue* | 4 | 1 | 1 | - |
|  | *Fever* | 1 | 1 | 1 | - |
|  | *Gait Disturbance* | 1 | - | - | - |
|  | *Malaise* | 1 | - | - | - |
|  | *Memory Loss* | 1 | - | - | - |
|  | *Non-Cardiac Chest Pain* | 1 | - | - | - |
|  | *Pain* | 1 | - | - | - |
|  | *Right Upper Quadrant Tenderness* | 1 | - | - | - |
| *Hepatobiliary disorders* | *Cholecystitis* | 1 | - | - | - |
| *Infections and infestations* | *Upper Respiratory Infection* | 1 | - | - | - |
|  | *Urinary Tract Infection* | - | - | 1 | - |
| *Injury, poisoning and procedural complications* | *Bruising* | 1 | - | - | - |
|  | *Fall* | - | - | 1 | - |
|  | *Fracture* | - | 1 | 1 | - |
| *Investigations* | *Alanine Aminotransferase Increased* | 1 | - | 1 | 1 |
|  | *Alkaline Phosphatase Increased* | - | 1 | - | - |
|  | *Aspartate Aminotransferase Increased* | - | - | 2 | 1 |
|  | *Blood Bilirubin Increased* | - | 1 | - | - |
|  | *CPK Increased* | 1 | - | - | - |
|  | *Creatinine Increased* | 2 | 1 | - | - |
|  | *Platelet Count Decreased* | 1 | - | - | - |
|  | *Serum Amylase Increased* | - | 1 | 1 | - |
|  | *Weight Loss* | 1 | - | - | - |
|  | *White Blood Cell Decreased* | 1 | - | - | - |
| *Metabolism and nutrition disorders* | *Anorexia* | 6 | 1 | - | - |
|  | *Dehydration* | 1 | 2 | - | - |
|  | *Hypoalbuminemia* | 3 | - | 1 | - |
|  | *Hypocalcemia* | 1 | - | - | - |
|  | *Hypokalemia* | 1 | - | - | - |
|  | *Hypomagnesemia* | 1 | - | - | - |
|  | *Hyponatremia* | 2 | - | - | 1 |
| *Musculoskeletal and connective tissue disorders* | *Generalized Muscle Weakness* | 1 | - | - | - |
|  | *Muscle Wasting* | - | 1 | - | - |
|  | *Pain In Extremity* | - | 1 | - | - |
| *Nervous system disorders* | *Dizziness* | 3 | - | - | - |
|  | *Dysgeusia* | - | 1 | - | - |
|  | *Tremor* | 1 | - | - | - |
| *Psychiatric disorders* | *Agitation* | 1 | - | - | - |
|  | *Anxiety* | 1 | 1 | - | - |
|  | *Confusion* | - | 1 | - | - |
|  | *Depression* | - | 1 | - | - |
| *Renal and urinary disorders* | *Acute Kidney Injury* | 1 | - | - | - |
|  | *Hematuria* | 3 | - | - | - |
|  | *Urine Discoloration* | 1 | - | - | - |
| *Reproductive system and breast disorders* | *Vaginal Inflammation* | 1 | - | - | - |
| *Respiratory, thoracic and mediastinal disorders* | *Cough* | 2 | 1 | - | - |
|  | *Dyspnea* | 2 | - | 1 | - |
|  | *Hypoxia* | - | - | 1 | - |
|  | *Productive Cough* | 1 | - | - | - |
| *Skin and subcutaneous tissue disorders* | *Alopecia* | 2 | - | - | - |
|  | *Dry Skin* | 1 | - | - | - |
|  | *Ecchymoses On Abdomen* | 1 | - | - | - |
|  | *Fissures On Hands* | 1 | - | - | - |
|  | *Head Laceration* | 1 | - | - | - |
|  | *Pruritus* | 4 | - | 1 | - |
|  | *Rash Acneiform* | 4 | 3 | - | - |
|  | *Rash Maculo-Papular* | 2 | 1 | 1 | - |
| *Vascular disorders* | *Hot Flashes* | 1 | - | - | - |
|  | *Hypotension* | 1 | 1 | - | - |
|  | *Thromboembolic Event* | - | - | 1 | - |
|  | *Venous Stasis* | 1 | - | - | - |
