# Supplementary material for: A Phase II Study of ERK Inhibition by Ulixertinib (BVD-523) in Metastatic Uveal Melanoma
Source: Cancer Res Commun. 2024 May 21;4(5):1321–7. doi: 10.1158/2767-9764.CRC-24-0036 (PMC11107576; doi:10.1158/2767-9764.CRC-24-0036)
Supplement: Supplementary table 3 — Adverse Events/toxicities felt to be at least possibly related to drug. [file crc-24-0036-s03.docx]

**Supplementary Table 3 – Adverse Events/Toxicities (at least possibly related)**

|  | | *Toxicity Grade CTCAE v4.0* | | | |
| --- | --- | --- | --- | --- | --- |
|  |  | *1* | *2* | *3* | *4* |
|  |  | *N* | *N* | *N* | *N* |
| *Toxicity Category CTCAE v4.0* | *Toxicity Description CTCAE v4.0* | - | - | 1 | - |
| *Blood and lymphatic system disorders* | *Anemia* |  |  |  |  |
|  | *Eosinophilia* | 1 | - | - | - |
| *Eye disorders* | *Blurred Vision* | 1 | - | - | - |
|  | *Floaters* | 1 | - | - | - |
|  | *Halos And Glowing Around Lights* | 1 | - | - | - |
|  | *Scotoma* | 1 | - | - | - |
| *Gastrointestinal disorders* | *Abdominal Pain* | 2 | - | - | - |
|  | *Anal Ulcer* | - | 1 | - | - |
|  | *Constipation* | 1 | - | - | - |
|  | *Diarrhea* | 9 | 1 | - | - |
|  | *Epigastric Pain* | 1 | - | - | - |
|  | *Esophageal Stenosis* | 1 | - | - | - |
|  | *Mucositis Oral* | 1 | - | - | - |
|  | *Nausea* | 3 | 2 | - | - |
|  | *Rectal Ulcer* | 1 | - | - | - |
|  | *Vomiting* | 1 | 1 | - | - |
| *General disorders and admin site conditions* | *Fatigue* | 3 | - | - | - |
|  | *Fever* | 1 | 1 | - | - |
|  | *Malaise* | 1 | - | - | - |
| *Investigations* | *Alanine Aminotransferase Increased* | - | - | 1 | 1 |
|  | *Aspartate Aminotransferase Increased* | - | - | 1 | 1 |
|  | *Creatinine Increased* | 2 | 1 | - | - |
|  | *Serum Amylase Increased* | - | - | 1 | - |
|  | *White Blood Cell Decreased* | 1 | - | - | - |
| *Metabolism and nutrition disorders* | *Anorexia* | 1 | 1 | - | - |
|  | *Hypoalbuminemia* | 2 | - | - | - |
|  | *Hypocalcemia* | 1 | - | - | - |
|  | *Hyponatremia* | 1 | - | - | 1 |
| *Nervous system disorders* | *Dizziness* | 1 | - | - | - |
|  | *Tremor* | 1 | - | - | - |
| *Reproductive system and breast disorders* | *Vaginal Inflammation* | 1 | - | - | - |
| *Respiratory, thoracic and mediastinal disorders* | *Cough* | 1 | - | - | - |
| *Skin and subcutaneous tissue disorders* | *Alopecia* | 2 | - | - | - |
|  | *Fissures On Hands* | 1 | - | - | - |
|  | *Pruritus* | 3 | - | 1 | - |
|  | *Rash Acneiform* | 4 | 3 | - | - |
|  | *Rash Maculo-Papular* | 2 | 1 | 1 | - |
| *Vascular disorders* | *Hypotension* | 1 | - | - | - |
